# Comparison of membrane affinity-based method with size-exclusion chromatography for isolation of exosome-like vesicles from human plasma

**DOI:** 10.1186/s12967-017-1374-6

**Published:** 2018-01-09

**Authors:** Ruzena Stranska, Laurens Gysbrechts, Jens Wouters, Pieter Vermeersch, Katarzyna Bloch, Daan Dierickx, Graciela Andrei, Robert Snoeck

**Affiliations:** 1 Laboratory of Virology and Chemotherapy, Rega Institute for Medical Research, KU Leuven – University of Leuven, 3000 Leuven, Belgium; 2 Department of Oncology, Laboratory of Lipid Metabolism and Cancer, KU Leuven – University of Leuven, 3000 Leuven, Belgium; 3 Department of Hematology, University Hospitals Leuven, 3000 Leuven, Belgium; 4 Department of Oncology, Laboratory of Experimental Hematology, KU Leuven – University of Leuven, 3000 Leuven, Belgium; 5 Clinical Department of Laboratory Medicine, University Hospitals Leuven, 3000 Leuven, Belgium; 6 Department of Cardiovascular Sciences, KU Leuven – University of Leuven, 3000 Leuven, Belgium

**Keywords:** Extracellular vesicles, Exosomes-like vesicles, Biomarkers, Size-exclusion chromatography, exoEasy kit, Plasma

## Abstract

**Background:**

Plasma extracellular vesicles (EVs), especially exosome-like vesicles (ELVs), are being increasingly explored as a source of potential noninvasive disease biomarkers. The discovery of blood-based biomarkers associated with ELVs requires methods that isolate high yields of these EVs without significant contamination with highly abundant plasma proteins and lipoproteins. The rising interest in blood-based EV-associated biomarkers has led to the rapid development of novel EV isolation methods. However, the field suffers from a lack of standardization and often, new techniques are used without critical evaluation. Size exclusion chromatography (SEC) has become the method of choice for rapid isolation of relatively pure EVs from plasma, yet it has technical limitations for certain downstream applications. The recently released exoEasy kit (Qiagen) is a new membrane affinity spin column method for the isolation of highly pure EVs from biofluids with the potential to overcome most of the limitations of SEC.

**Methods:**

By using multiple complementary techniques we assessed the performance of the exoEasy kit in isolating ELVs from 2 ml of human plasma and compared it with the SEC qEV column (Izon Science).

**Results:**

Our data show that exoEasy kit isolates a heterogenous mixture of particles with a larger median diameter, broader size range and a higher yield than the SEC qEV column. The exclusive presence of small RNAs in the particles and the total RNA yield were comparable to the SEC qEV column. Despite being less prone to low density lipoprotein contamination than the SEC qEV column, the overall purity of exoEasy kit EV preparations was suboptimal. The low particle-protein ratio, significant amount of albumin, very low levels of exosome-associated proteins and propensity to triglyceride-rich lipoprotein contamination suggest isolation of mainly non-ELVs and co-isolation of plasma proteins and certain lipoproteins by the exoEasy kit.

**Conclusions:**

We demonstrate that performance of exoEasy kit for the isolation of ELVs for biomarker discovery is inferior to the SEC qEV column. This comprehensive evaluation of a novel EV isolation method contributes to the acceleration of the discovery of EV-associated biomarkers and the development of EV-based diagnostics.

**Electronic supplementary material:**

The online version of this article (10.1186/s12967-017-1374-6) contains supplementary material, which is available to authorized users.

## Background

The identification of extracellular vesicle (EV)-associated biomarkers is crucially dependent on methods that allow the isolation of EVs without contaminating plasma proteins and lipoproteins, yield a sufficient quantity of EVs for downstream molecular analysis, and are reproducible, efficient and easy to perform.

Isolation of EVs from plasma is prone to encounter several obstacles related to high plasma viscosity, high lipid and protein content and the presence of platelets. Frequently, the sample size is a limiting factor too. Currently, standardized protocols for isolation of EVs from plasma are missing.

The most popular method for EV isolation, differential ultracentrifugation, suffers from several limitations (impurities, EV aggregation, decreased integrity of EVs) and impracticalities (long turnaround time, specialized equipment) [1, 2]. The growing interest in blood-based EV-associated biomarkers in recent years has led to the development of novel EV isolation methods that could be well suited for clinical research. Despite the growing number of these methods, the field suffers from a lack of standardization and often, new techniques are used without detailed comparative analysis.

Size exclusion chromatography (SEC) has been shown to perform well in separating EVs from contaminating plasma proteins and high density lipoproteins (HDL) [1, 3, 4] and has been successfully used for small scale analysis of EVs from clinical samples [4, 5]. Despite becoming the method of choice for isolation of relatively uncontaminated EVs from plasma, SEC has several technical and practical limitations. SEC only permits efficient isolation of EVs larger than the pore size of the matrix used (i.e. 70 nm for CL-2B Sepharose). Although free of HDLs, EV-rich fractions can still contain a small amount of other lipoproteins such as chylomicrons (100–600 nm) and very low density lipoproteins [VLDL (30–80 nm)] [4, 6, 7]. Although the sample processing time is short (20 min) compared to differential ultracentrifugation, SEC still requires considerable hands-on time for the preparation of the isolation column (if homemade), washing and (re)equilibration. In addition, manual collection of fractions may introduce operator-based variability, especially affecting the purity of the fractions. Other limitations of SEC include the relatively low vesicle yield and the dilution of a purified sample which requires an additional concentrating step that may result in a yield drop [3].

Recently, a new membrane affinity spin column method for the isolation of highly pure EVs from biofluids was released (exoEasy kit from Qiagen) [8] that appears to have the potential to overcome many of the limitations of SEC. In addition, EV isolation can easily be coupled with RNA extraction directly from the EVs bound to the column membrane (exoRNeasy Serum/Plasma kit), hence offering a simplified workflow for downstream analysis of the RNA content of EVs, which could facilitate clinical research into novel EV-associated RNA biomarkers.

Here we have compared the large sample volume version of the exoEasy kit, the exoEasy Maxi kit, with the SEC using qEV 10 ml columns (Izon Science) for isolation of EVs from 2 ml of human plasma. We have focused our analysis particularly on ELVs, the clinically most relevant EV subset. We have evaluated the yield, size distribution and purity of isolated EVs as well as their RNA content including size range and yield.

Our data show that the exoEasy kit isolates a heterogeneous mixture of particles with a larger median diameter, broader size range and a higher yield than the SEC qEV column. The exclusive presence of small RNA in the particles and the total RNA yield were comparable between the kits. Despite being less prone to low density lipoprotein (LDL) contamination than the SEC qEV column, the low particle-protein ratio, significant amount of albumin, very low levels of exosome-associated proteins and propensity to triglyceride-rich lipoprotein contamination suggest isolation of mainly non- ELVs and co-isolation of plasma proteins and certain lipoproteins by the exoEasy kit.

## Methods

### Sample collection and preparation of platelet-free plasma (PFP)

Two groups of six healthy anonymous donors and three lymphoma patients were included in the study. Blood was drawn from non-fasting donors into K_2_ EDTA tubes (BD Vacutainer) and processed within 2 h of blood draw. It was centrifuged at 2500×*g* for 15 min at 20 °C. Cell-free, platelet poor plasma was collected and subjected to the second centrifugation under the same conditions. The supernatant was finally centrifuged at 13,000×*g* for 5 min and the resulting PFP was filtered through a 0.22 µm filter, aliquoted and stored at − 80 °C [9]. Prior to use, plasma was quickly thawed in a water bath at 37 °C and clarified by centrifugation at 10,000×*g* for 20 min to remove apoptotic bodies and microvesicles. The first set of six plasma samples from healthy donors was processed as described above. For the second set of plasma samples, collected from additional six healthy donors and three lymphoma patients and used primarily for determination of total lipid levels of plasma EVs, plasma ultrafiltration and high-speed centrifugation were omitted.

### EV isolation from plasma using SEC qEV columns

After rinsing the columns with PBS, 2 ml of PFP were applied on top of a qEV column (Izon Science) and 0.5 ml fractions were collected. Four EV-rich fractions (7–10) were pooled and either analyzed directly (see below) or concentrated using an Amicon Ultra-4 10 kDa centrifugal filter device (Merck Millipore).

### EV isolation from plasma using exoEasy kit (Qiagen)

This was performed from 2 ml of PFP according to the manufacturer’s protocol with modifications described in Enderle et al. [8]. EV eluates were either analyzed directly (see below) or concentrated as described above. EV eluates from both the SEC qEV columns and the exoEasy kit were aliquoted in low protein binding tubes and single use aliquots were stored at − 80 °C.

### Total protein quantification and Western blot analysis

Proteins were extracted from concentrated pooled EV-rich SEC qEV fractions or exoEasy kit eluates using a lysis buffer (1% NP40, 1 mM EDTA) with a protease inhibitor cocktail (Roche). Samples were vortexed and lysed on ice for 15 min. The total protein content of EVs was measured by Pierce BCA Protein Assay Kit (Thermo Scientific). To assess the purity of EV preparations, a ratio of number of particles to micrograms of protein was calculated [10]. The presence of EV-enriched proteins as well as the absence of endoplasmatic reticulum (ER) markers and plasma proteins was determined by Western blotting in 10 µg of lysates using the following antibodies: syntenin-1 (gift from P. Zimmermann), Tsg101 (612697, BD Biosciences), CD63 (556019, BD Biosciences), CD81 (sc-166028, Santa Cruz), calnexin (sc-2679, Santa Cruz) and albumin (4929, Cell signaling).

### Total lipid quantification

Lipid content of EV preparations was determined by sulpho-vanilin assay using the Lipid Quantification Kit (Cell Biolabs, San Diego, USA) under conditions optimized for EV analysis [6].

### Determination of plasma lipoproteins

Levels of plasma triglycerides, total cholesterol, HDL, LDL, non-HDL cholesterol, apoA1 and apoB were determined in a clinical diagnostic laboratory of University Hospitals Leuven on a Roche Cobas 8000 chemistry analyzer.

### Transmission electron microscopy (TEM); negative staining

Aliquots from pooled EV-rich SEC fractions or exoEasy kit eluates were deposited onto formvar-coated 400 mesh copper grids for 7 min at room temperature and thereafter stained with 1.75% uranyl acetate. The grids were observed using a transmission electron microscope JEM 1400 (Jeol Ltd.).

### Nanoparticle Tracking Analysis (NTA)

This was performed using a NanoSight LM10 instrument (Malvern Instruments Ltd.). Aliquots from pooled EV-rich SEC fractions or exoEasy kit eluates were diluted in filtered PBS. Six videos of 30 s were captured for each sample.

### RNA isolation from EVs

Total RNA was isolated from concentrated pooled EV-rich SEC fractions or directly from exoEasy kit columns following the protocol of the exoRNeasy Serum/Plasma Kit (Qiagen). The protocol was optimized to increase the yield by addition of glycogen (5 µg/ml), double extraction of the aqueous phase [11] and double elution of RNA. RNA yield and size range were analyzed on an Agilent 2100 Bioanalyzer using the RNA 6000 Pico Kit and the Small RNA Kit (Agilent Technologies).

### Statistical analysis

Data were analyzed with the GraphPad Prism software using Wilcoxon matched-pairs signed rank test with p values * < 0.05 and ** < 0.01. Data are presented as mean ± SEM.

## Results

### ExoEasy kit isolates a highly heterogeneous mix of particles from plasma including EVs, proteins and lipoproteins

**Fig. 1:**
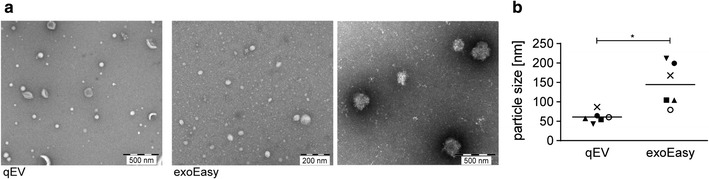
TEM analysis of EVs isolated by exoEasy kit and SEC qEV columns. Negative staining using uranyl acetate. **a** Representative images of EV isolated from the same donor. ExoEasy samples were diluted 1/20. Magnification ×5000 and ×10,000; scale bar 500 nm (qEV and right image of exoEasy) and 200 nm (left image of exoEasy). **b** Diameter of isolated particles. A total of 50 particles were analyzed in at least three independent images per donor. Particles with diameter of > 20 nm were measured. (mean ± SEM, n = 6; *p < 0.05, Wilcoxon matched-pairs signed rank test)

The TEM analysis of pooled fractions 7–10 from the SEC qEV columns showed enrichment of exosome-like particles with a round or cup-shaped appearance, typical for this type of EM analysis (Fig. 1a). Apart from ELVs that can range in size from 30 to 150 nm, we also observed smaller plasma lipoprotein particles [low density lipoproteins (LDL)] with a diameter of around 25 nm that were not efficiently removed by the qEV column. Lipoprotein contamination of SEC EV preparations has been previously reported [4].

Plasma EV preparations using the exoEasy kit contained high concentration of particles (proteins and lipoproteins) and had to be diluted up to 100 times to enable observation of individual particles. We found a highly heterogeneous mixture of particles in these eluates, illustrated by multiple TEM images (Fig. 1a), with a diameter ranging from 23 to 511 nm (a median of 150 nm, Fig. 1b). ELVs were observed, but much less frequently than in SEC qEV eluates. Small (~ 25 nm) particles with round morphology, likely lipoproteins, were also present. In stark contrast to EVs prepared by the SEC qEV column, we also noted a substantial presence of proteins (small irregular structures; Fig. 1a). Finally, we observed large non-spherical particles with a size exceeding 200 nm. These structures were present frequently and consistently in repeated experiments in the samples from all six healthy donors of the first group, while they were absent from the kit elution buffer. The irregular morphology of these particles was suggestive of protein aggregates [12]. The identity of these particles is yet unknown.

**Fig. 2:**
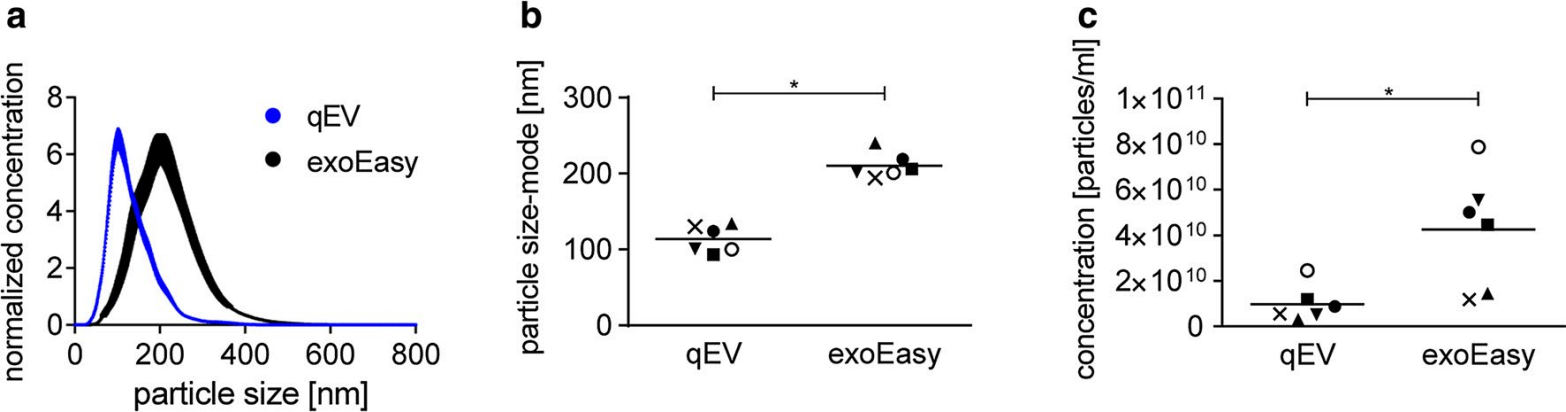
Nanoparticle tracking analysis of EVs isolated by exoEasy kit and SEC qEV columns. **a** Representative size distribution profiles of particles isolated from the same donor. Normalized concentration of samples is shown. **b** Mode diameter of particles and **c** concentration of particles. (mean ± SEM, n = 6; *p < 0.05, **p < 0.01, Wilcoxon matched-pairs signed rank test)

NTA of EVs isolated by the SEC qEV columns showed the presence of particles with a size distribution corresponding to the size range of ELVs; with an average mode diameter of 117 nm. Particles isolated using the exoEasy kit showed a broader size distribution and the principal population was significantly larger; with a mode diameter of 210 nm (Fig. 2a, b). These results largely agree with those of the TEM analysis confirming that the majority of the EVs isolated by affinity spin columns was significantly larger than the particles isolated by the SEC qEV columns.

**Fig. 3:**
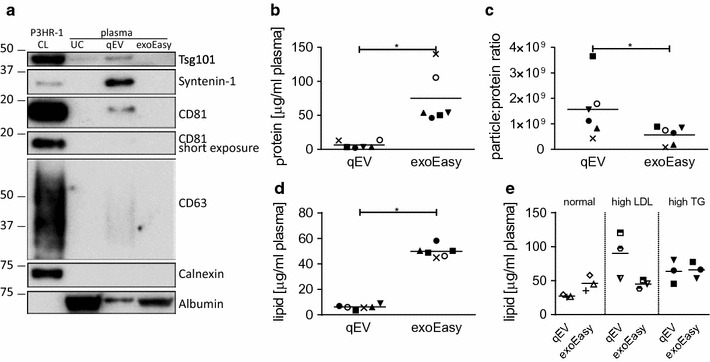
Protein analysis of EVs isolated by exoEasy kit and SEC qEV columns. **a** Western blotting analysis of proteins considered as exosome markers, non-exosomal proteins and albumin. As controls, cell lysate (CL) of P3HR-1 lymphoblastoid cell line and plasma ELVs prepared by ultracentrifugation (UC; based on protocol described by Thery et al [30]) are shown. Representative donor of six analyzed is shown. **b** Total protein content. **c** Particles to protein ratio and **d** total lipid content of EV preparations from an original set of plasma samples from six healthy donors, as described in “Methods”. (mean ± SEM, n = 6; *p < 0.05, **p < 0.01, Wilcoxon matched-pairs signed rank test). **e** Total lipid content of EV preparations from a second set of plasma samples from six healthy donors and three lymphoma patients, as described in Methods. (mean ± SEM, n = 3)

The NTA also showed a significantly higher number of particles isolated by the exoEasy kit compared to the SEC qEV columns (Fig. 2c). This result was expected given the affinity of the exoEasy column membrane for all EV types and is in line with the broader particle size distribution profile detected by NTA. However, we did not observe a corresponding increase in the enrichment of exosomal markers in exoEasy kit preparations (Fig. 3a) which would suggest a non-exosomal origin of the majority of the isolated particles.

### Suboptimal purity of exoEasy kit EV preparations

We measured the amount of protein present in the EV samples to evaluate the purity of the isolated EVs. The eluates from the exoEasy kit contained significantly higher concentrations of total protein than the pooled EV-rich fractions from the SEC qEV columns (Fig. 3b). The more than tenfold difference in the protein content suggests co-isolation of contaminating plasma proteins and/or lipoproteins by exoEasy kit.

To further assess the purity, Western blot analysis was performed with equal protein loading which allows to directly evaluate the purity of the EV samples by comparing the enrichment of proteins regarded as exosomal markers and the contamination with ER or plasma proteins (Fig. 3a). A recent in depth proteomic analysis of EV content by Kowal et al. has re-evaluated the so-called exosomal markers [13]. From the proposed updated set of exosome-specific proteins, which include syntenin-1, TSG101, ADAM10 and EDH4, we have found syntenin-1 to be highly enriched in EVs isolated by the SEC qEV columns, while it was almost undetectable in EVs isolated by the exoEasy kit. A weak syntenin-1 band was detected only after a long exposure of the blots (data not shown). TSG101 was enriched in EVs isolated by qEV columns, but it was undetectable in EVs isolated by exoEasy kit. Kowal et al. further pointed out that the tetraspanin CD63 is expressed, apart from in ELVs, also in apoptotic bodies and hence is less exosome specific, while the expression of CD81 was more restricted, with a significantly higher presence in ELVs compared to other EVs [13]. Accordingly, we detected an enrichment of CD81 in EVs isolated from the SEC qEV column but not in those isolated from the exoEasy kit. The same result was obtained for CD63, with undetectable levels found in EVs from the exoEasy kit. Our results further show that EVs isolated by both kits are not contaminated with endoplasmic reticulum membranes components, as indicated by the absence of calnexin. Albumin, the most abundant plasma protein, was readily detectable in exoEasy kit EVs, with an enrichment over five times higher compared to the SEC qEV column EVs. Indeed, residual albumin, albeit in lower quantities, was also detected in EVs prepared by the SEC qEV columns, as observed previously [14].

Another measure of particle purity, the particle-protein ratio [10], was significantly lower for the exoEasy kit than for SEC qEV samples, indicating co-isolation of proteins in exoEasy kit samples (Fig. 3c). The high protein content, the low particle-protein ratio together with the undetectable/low levels of exosome markers suggest a contamination of exoEasy EV preparations with non-EV plasma proteins and lipoproteins.

Since detailed comparative analysis of lipoproteins in EV preparations from the exoEasy kit and the SEC qEV column was not feasible, due to limitations in plasma sample volume, we opted for an alternative analysis, which consisted in determining the total lipid content of EV preparations by a sulfophosphovanilin (SPV) assay [15]. In this context, the total lipid content reflects not only the total content of the EV-derived lipids but also the total level of lipoprotein contamination of the EV preparations. Analysis of EVs prepared from plasma of six healthy donors from the first patient group showed low concentrations of total lipids in the SEC qEV preparations, not exceeding 10 ng per ml of plasma. In contrast, the EVs isolated by exoEasy kit had significantly higher total lipid content, with a mean eightfold difference compared to the SEC qEV-derived EVs (Fig. 3d). This result correlates with the higher protein content of exoEasy EVs compared to EVs isolated by the SEC qEV column (Fig. 3b) and likely reflects both the presence of larger particles as well as the co-isolation of plasma lipoproteins by exoEasy kit. As shown in Additional file 1: Table S1, concentrations of lipids and lipoproteins in the healthy donors’ plasma used for EV isolation were all in a normal range [16]. Thus, these data served as reference for further assessment of the impact of specific lipoprotein classes on the level of lipoprotein contamination of EV preps. To accomplish this, we used SPV assay to analyze an additional set of three plasma samples with high levels of LDLs (derived from healthy donors), three samples with high levels of triglycerides (derived from three lymphoma patients) along with three samples from healthy donors with normal levels of all plasma lipoproteins (Additional file 2: Table S2). To assess the specific effects of these plasma constituents with a greater sensitivity, plasma was processed without ultrafiltration and high-speed centrifugation prior to EV isolation. Analysis of EVs isolated from the plasma of three healthy donors showed again higher total lipid levels in exoEasy kit EV preparations compared to those prepared by SEC qEV columns (Fig. 3e). The mean total lipid levels of SEC qEV preparations were increased compared to those from the first set of healthy donor plasma (Fig. 3d). This result correlated with the levels of plasma lipids of these three healthy donors, which were elevated (except for triglycerides) albeit within the normal range (Additional file 2: Table S2), compared to the first healthy donor group (Additional file 1: Table S1). The fact that the total lipid levels of the exoEasy kit preparations were not affected by the change in lipoprotein levels likely indicates that the exoEasy kit is less prone to co-isolation of these lipoproteins compared to the SEC qEV column.

High levels of plasma LDLs in the 3 healthy donors analyzed (Additional file 2: Table S2) did not affect the total lipid content of EVs prepared by the exoEasy kit, while it resulted in a dramatic increase in the lipid concentrations in EV preparations from the SEC qEV columns (Fig. 3e). Increased levels of triglycerides in the plasma of 3 lymphoma patients (Additional file 2: Table S2) led to an increase in the total lipid levels in EVs prepared by both the exoEasy kit and the SEC qEV columns. Collectively, these results show that the total lipid content of the exoEasy kit preparations is higher than that of the SEC qEV columns. This might reflect both the capacity of the exoEasy kit to isolate wider range of vesicles than the SEC qEV columns, as shown in Fig. 2a, as well as the presence of lipoproteins in EV preparations. The exoEasy kit does not seem to co-isolate LDLs, which are present in high levels in SEC qEV preparations. Nevertheless, the purity of exoEasy EV preparations seems to be compromised by co-eluted triglyceride-rich lipoproteins (Fig. 3e).

### exoEasy kit EV preparations contain exclusively small RNAs with a yield comparable to SEC

**Fig. 4:**
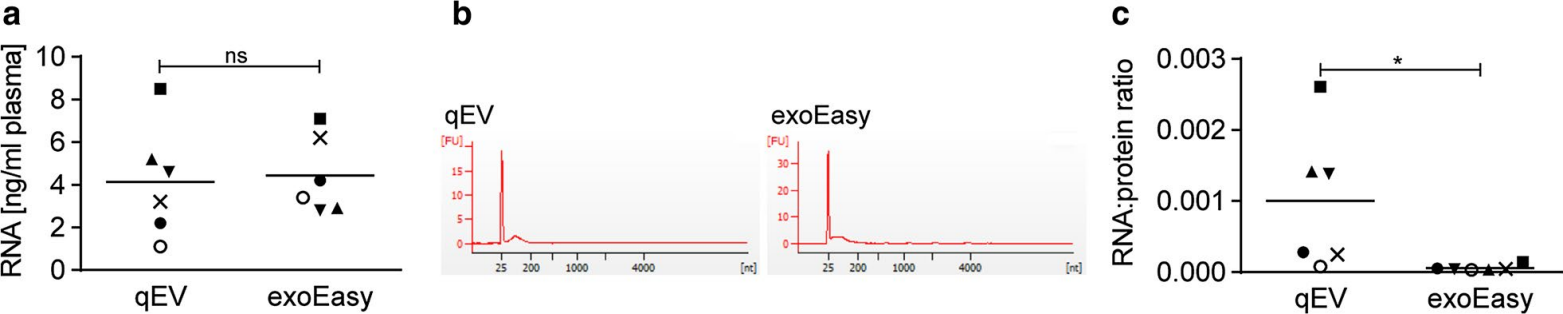
RNA analysis of EVs isolated by exoEasy kit and SEC qEV columns. **a** RNA concentration. **b** Representative bioanalyzer profiles of RNA isolated from the same donor and analyzed by RNA 6000 Pico Kit; the y-axis shows fluorescence units (FU) and the x-axis the nucleotide length (nt) of the RNA. Peaks at 25 nt is an internal standard. **c** Ratio of total amount of RNA (µg) to total amount of protein (µg). (mean ± SEM, n = 6; *p < 0.05, ns; not significant, Wilcoxon matched-pairs signed rank test)

As ELVs are considered an important source of RNA-based biomarkers, we assessed the total RNA content of EVs by Bioanalyzer. We found similar RNA recovery by both kits, albeit with a slightly lower yield for the SEC qEV columns (Fig. 4a). The low RNA yields detected here, with a median of ~ 3.8 ng and a range from 2.8 to 7.1 ng per ml of plasma are typical for EVs [17–19] and were reported previously for exoEasy kit [8]. The Bioanalyzer profiles of isolated RNA showed the presence of small RNAs and the virtual absence of rRNA for both methods (Fig. 4b). This profile was previously shown to be characteristic for ELVs [20, 21] and is consistent with the data from the first evaluation of this kit [8]. We also calculated the RNA to protein ratio, which was previously found to be higher in ELVs than in microvesicles suggesting more RNA associated with ELVs [20]. This ratio was significantly higher for EVs isolated by the SEC qEV columns than for EVs isolated by the exoEasy kit (Fig. 4c) suggesting that potentially more non-ELVs are isolated by the exoEasy kit.

## Discussion

The growing interest in molecules carried by EVs as potential circulating biomarkers has spurred a lot of research and development into the methods for isolation of plasma EVs, especially ELVs. The isolation of a high yield of high-purity ELVs from plasma is technically challenging especially due to its high density and viscosity and the complex composition [different types of vesicles, proteins, ribonucleoproteins (RNP), lipoproteins]. The methods and protocols to achieve this goal are thus under constant development.

Recently several novel methods have emerged in the form of a kit that enable an easy and fast isolation of EVs from plasma. Among EV isolation methods potentially suitable for an EV biomarker discovery we identified exoEasy kit (Qiagen), based on its primary evaluation by Enderle et al. [8], to be a potentially promising, faster and easier-to-use alternative to SEC, the current method of choice. EV isolation by the membrane affinity spin column of exoEasy kit is based on a universal biochemical feature specific to EVs. Other particles, such as plasma proteins, lipoproteins or RNP complexes that often contaminate EVs prepared by classical methods, should not be co-isolated. The broader size range expected for EVs isolated by the exoEasy kit compared to the lower particle size limit of 70 nm for EVs isolated by SEC qEV columns could improve the detection of low abundance biomarkers and/or enable detection of small size EV-based biomarkers. Different column sizes allow the isolation of EVs from plasma volumes ranging from 0.1 to 4 ml. The option to extract RNA directly from column-bound EVs (exoRNeasy Serum/Plasma kit) is another feature that makes this kit attractive for potential clinical use. The time required for the purification of EVs using the exoEasy kit (25 min) is largely the same as required for the SEC qEV columns without the additional hands-on time for qEV column washing and re-equilibration. ExoEasy kit columns cannot be re-used, but enable processing up to 4 ml of plasma. Although plasma volumes processed by a 10 ml SEC qEV column vary in literature, only 0.5 ml is recommended by the manufacturer. The SEC qEV columns however, can be re-used up to five times thus enabling processing of up to 2.5 ml of plasma.

In this report we provide a comprehensive assessment of the exoEasy kit for the isolation of EVs, particularly ELVs, from human plasma, focusing on its comparison to the currently very popular SEC qEV columns. We used multiple complementary techniques for this purpose [19, 22].

The results of TEM and NTA analyses indicate that exoEasy kit isolation results in a highly concentrated sample containing a heterogeneous mix of particles that differ greatly in size and shape. Both analysis methods show that the majority of particles isolated by affinity spin columns were significantly larger than particles isolated by the SEC qEV columns, with a diameter exceeding the typical size range of ELVs (30–150 nm) [23]. The difference in the EV sizes measured by NTA and TEM were expected given that particles are analyzed in a hydrated and desiccated state, respectively. The hydrodynamic diameter of ELVs was reported to be up to three times larger than the geometric one [24]. In line with our data, others also have detected the presence of larger particles in eluates from exoEasy kit when using TEM and light scattering methods as a readout [25]. These larger particles were attributed to the kit elution buffer. Our NTA analysis of elution buffer however did not confirm these findings.

Previous studies have highlighted the differences in EV diameter between patients with classical Hodgkin lymphoma and healthy donors [5, 26] with patients displaying particles with a smaller diameter. The propensity of the exoEasy kit to isolate larger vesicles might lead to a suboptimal isolation of the population of small EVs from the patients’ plasma and might thus affect the subsequent biomarker analysis. Similarly, the SEC qEV column cut-off is 70 nm and thus it is also likely to inefficiently isolate smaller EVs. While here we focused primarily on evaluation of the exoEasy kit’s the capacity to isolate ELVs from plasma, the isolation of a broad range of vesicles by the exoEasy kit could be useful in certain applications.

A high particle concentration was detected by both NTA and TEM in exoEasy eluates, however it was not reflected by the number of ELVs observed by TEM. Instead, the presence of proteins and large size particles was readily detected upon sample dilution. Given that NTA does not differentiate between vesicles and non-vesicular particles/aggregates, the higher particle concentrations detected in EV preparations from exoEasy kit could reflect the presence of RNP complexes, protein aggregates or lipoproteins.

Lipoproteins are a major subcellular particle subset of plasma [27], thus it is not surprising that they are frequently found in EV preparations from plasma. The presence of contaminating lipoproteins in EVs could potentially affect certain downstream molecular analyses For example HDLs were identified as circulating miRNAs carriers [28] Currently, no stand-alone EV isolation method results in a complete removal of plasma lipoproteins. SEC efficiently removes contaminating proteins (< 1%) and HDLs (< 5%) from plasma EVs [3–5] however recent reports have shown that lipoproteins, especially LDLs (~ 25 nm, but also aggregates 100–600 nm) might co-isolate in exosomal fractions retrieved from the SEC qEV columns [3, 6, 27]. Indeed, we have observed small spherical particles in our SEC qEV preparations with a diameter corresponding to that of LDL. In addition, we obtained evidence for co-isolation of LDLs and triglyceride-rich lipoproteins (chylomicrons, VLDL and their remnants) in SEC EV preparations based on the total lipid analysis of EVs from plasma of subjects with distorted plasma lipoprotein profiles. It is assumed that lipoproteins do not co-isolate with EVs during exoEasy kit-based EV isolation [8], however this was not previously excluded. Here we demonstrate that exoEasy kit is more effective in removing contaminating plasma LDLs from EV preparations than the SEC qEV columns, while still showing propensity to contamination with triglyceride-rich lipoproteins. Since our other observations including the presence of vesicles in the size range of VLDLs, a high protein content and a low particle-protein ratio could serve as additional indirect evidence for the presence of co-isolated lipoproteins, further evaluation of co-isolation of other lipoprotein classes is warranted.

In a previously published evaluation of the exoEasy kit only a single vesicle marker was used to detect the presence of EVs [8] and the purity of the EV preparations was not assessed. In addition, the manufacturer’s recommendation to concentrate the isolated EVs using a protein filter with ≤ 100 kDa pore size may mask the presence of contaminating plasma proteins. Focusing our protein analysis on a recently updated set of proteins characteristic of ELVs we confirmed the high enrichment of these markers, including syntenin-1, TSG101, and CD81 in samples from SEC qEV columns. In a stark contrast, however, these proteins were either undetectable or present only at very low levels in exoEasy kit samples. The presence of albumin indicates an insufficient purity of the EV preparation, as albumin has been shown not to be part of the EV proteome [29]. Albumin co-isolated with EVs from exoEasy kit samples at much higher levels compared to SEC and together with the low particle to protein ratio was another indicator of inferior purity of exoEasy kit EVs. We and others have detected low levels of albumin in vesicle-rich fractions eluted from SEC qEV columns [14]. We should point out that the pool of fractions 7–10 was used in this analysis. Using a pool limited to fractions 7–8/9 results in lower levels of contaminating albumin (manufacturer’s instructions). qEV columns contain the CL-2B Sepharose. Recently other matrices (Sepharose CL-4B or Sephacryl S-400) were shown to perform better in the separation of EVs from albumin [14].

## Conclusions

Here we demonstrate that the performance of the membrane affinity-based exoEasy kit is inferior to the SEC in isolating ELVs from human plasma at a quality required for identification of ELV-associated biomarkers. This study contributes to the rapidly growing EV biomarker field for which critical evaluation of novel EV isolation methods is essential.

## Additional files

**Additional file 1.** Lipid and lipoprotein composition of plasma from the first sample set (healthy donors).

**Additional file 2.** Lipid and lipoprotein composition of plasma from the second sample set (healthy donors and lymphoma patients).

## Abbreviations

EV: extracellular vesicles
SEC: size exclusion chromatography
HDL: high density lipoprotein
PFP: platelet-free plasma
ER: endoplasmatic reticulum
TEM: transmission electron microscopy
NTA: nanoparticle tracking analysis
LDL: low density lipoprotein
RNP: ribonucleoprotein
VLDL: very low density lipoprotein
TG: triglyceride
