# Supplementary material for: Comparison of membrane affinity-based method with size-exclusion chromatography for isolation of exosome-like vesicles from human plasma
Source: J Transl Med. 2018 Jan 9;16:1. doi: 10.1186/s12967-017-1374-6 (PMC5761138; doi:10.1186/s12967-017-1374-6)
Supplement: Supplementary file 1 — Additional file 1. Lipid and lipoprotein composition of plasma from the first sample set (healthy donors). [file 12967_2017_1374_MOESM1_ESM.docx]

**Table S1. Lipid and lipoprotein composition of plasma from the first sample set (healthy donors).**

| Lipid/lipoprotein | [mg/dl] |
| --- | --- |
| Triglycerides | 66 ± 8.0 |
| Total cholesterol | 114 ± 14.2 |
| HDL | 47 ± 6.8 |
| LDL cholesterol | 57 ± 9.3 |
| Non-HDL cholesterol | 68 ± 10.2 |
| Apo-A1 | 122 ± 15.4 |
| ApoB | 54 ± 6.0 |

Mean ± SEM is shown, N=6. Mean HDL and apo-A1 values are below the normal range for females.
