# Supplementary material for: Comparison of membrane affinity-based method with size-exclusion chromatography for isolation of exosome-like vesicles from human plasma
Source: J Transl Med. 2018 Jan 9;16:1. doi: 10.1186/s12967-017-1374-6 (PMC5761138; doi:10.1186/s12967-017-1374-6)
Supplement: Supplementary file 2 — Additional file 2. Lipid and lipoprotein composition of plasma from the second sample set (healthy donors and lymphoma patients). [file 12967_2017_1374_MOESM2_ESM.docx]

**Table S2. Lipid and lipoprotein composition of plasma from the second sample set (healthy donors and lymphoma patients).**

| Lipid/lipoprotein  [mg/dl] | Healthy donors  (normal levels) | Healthy donors  (high LDL) | Lymphoma patients  (high triglycerides) |  |
| --- | --- | --- | --- | --- |
| Triglycerides | 62 ± 10.8 | 129 ± 35.2 | **257 ± 63.5** |  |
| Total cholesterol | 173 ± 1.2 | **241 ± 23.3** | 170 ± 12.8 |  |
| HDL | 75 ± 16.7 | 79 ± 19.7 | *36 ± 9.4* |  |
| LDL cholesterol | 89 ± 17.2 | 135 ± 12.9 | 83 ± 10.4 |  |
| Non-HDL cholesterol | 98 ± 17.6 | 161 ± 4.7 | 135 ± 20.5 |  |
| Apo-A1 | 205 ± 39.6 | 220 ± 27.8 | 145 ± 33 |  |
| ApoB | 85 ± 13.4 | **123 ± 7.2** | 106 ± 16.7 |  |

Plasma from 3 healthy donors with normal plasma lipid levels, 3 healthy donors with elevated LDL markers and 3 lymphoma patients with elevated triglycerides. Mean ± SEM is shown. Values considered high and borderline high are in bold and underlined, respectively. Values below normal range are in italics.
